# RETRACTION: Screening of Key Modules and Key Genes in the Prevention of Skin Cancer Development Based on Gene Volcano Plot and WGCNA

**DOI:** 10.1111/srt.70152

**Published:** 2025-01-07

**RETRACTION**: R. Yuan, Y. Bai, and H. Du, “Screening of Key Modules and Key Genes in the Prevention of Skin Cancer Development Based on Gene Volcano Plot and WGCNA,” *Skin Research and Technology* 30, no. 7 (2024): e13873, https://doi.org/10.1111/srt.13873.

The above article, published online on 28 July 2024 in Wiley Online Library (wileyonlinelibrary.com), has been retracted by agreement between *Skin Research and Technology*; and John Wiley & Sons Ltd. The article was submitted as part of a guest‐edited special issue. Following publication, it has come to the attention of the journal that the article was accepted solely on the basis of compromised editorial handling and peer review processes. Furthermore, the article lacks a coherent narrative, resulting in it being incomprehensible to readers. Finally, its conclusions are not supported by the data presented, further undermining its validity.

Therefore, the decision to retract this article was taken.

